# The Characteristics Analysis of a Microfluid-Based EGFET Biosensor with On-Chip Sensing Film for Lactic Acid Detection

**DOI:** 10.3390/s22155905

**Published:** 2022-08-07

**Authors:** Po-Yu Kuo, Chun-Hung Chang, Wei-Hao Lai, Tai-Hui Wang

**Affiliations:** Graduate School of Electronic Engineering, National Yunlin University of Science and Technology, Douliu 64002, Taiwan

**Keywords:** extended gate field-effect transistor (EGFET), microfluid, ruthenium dioxide (RuO_2_), Lactic acid (LA), on-chip sensing window (OCSW)

## Abstract

In this research, a microfluid-based extended gate field-effect transistor (EGFET) biosensor with an on-chip sensing window (OCSW) was fabricated. The detection window was composed of six metal layers, and a ruthenium dioxide (RuO_2_) film was spattered on the surface and functionalized with lactase to detect lactic acid (LA). To detect LA in a more diversified way, a microfluidic system was integrated with the biosensor. Moreover, a special package was used to seal the sensing window and microfluidic tube and insulate it from other parts to prevent water molecule invasion and chip damage. The sensitivity analysis of the EGFET biosensor was studied by a semiconductor parameter analyzer (SPA). The static and dynamic measurements of the EGFET with sensing windows on a chip were analyzed. The sensing characteristics of the EGFET biosensor were verified by the experimental results. The proposed biosensor is suitable for wearable applications due to the advantages of its low weight, low voltage, and simple manufacturing process.

## 1. Introduction

**Figure 1:**
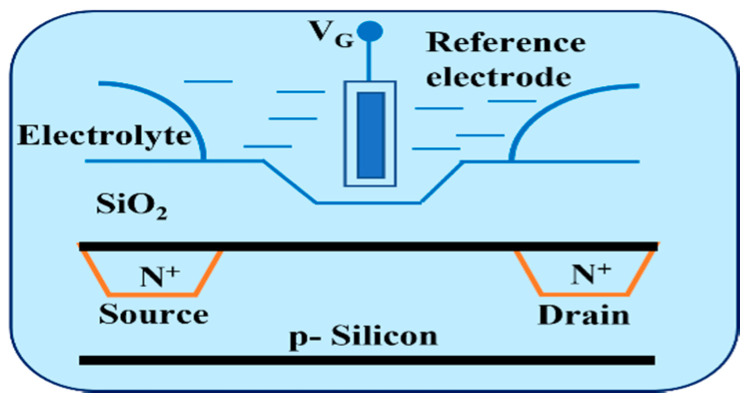
The structure of ISFET.

**Figure 2:**
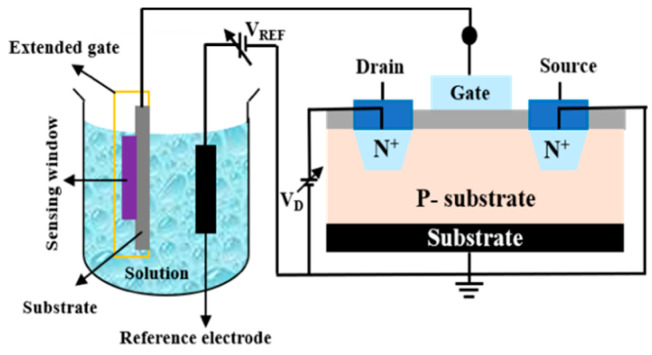
The basic cross-section of EGFET.

The metal oxide semiconductor field-effect transistor (MOSFET) is composed of a p–n junction and metal oxide semiconductor capacitor in which a gold oxygen capacitor is assembled into the transistor. In 1959, the team of D. Kahng and M. M. Atalla successfully developed the first MOSFET [1]. The MOSFET has become the most important device in the integrated circuit of semiconductor devices. Different types of MOSs with different structures and driving modes have been developed. MOS elements are usually treated with a semiconductor substrate, and the dielectric layer is formed by thermal oxidation with SiO_2_ on the surface, which can be successively implemented by ion implantation, photolithography, and etching processes. The metal is then stacked in a deposition manner to form a memory capacitance [2,3]. In 1970, the ion-sensitive field-effect transistor (ISFET) was proposed by P. Bergved [4]. The ISFET evolved from the MOSFET, and it is considered an electrochemical-sensing biosensor that is used to measure ion concentrations in solutions. This biosensor has an equally fundamental structure to MOSFET, but this element has no metal layer. Instead, it uses an ion-sensing film and reference electrodes. When the ISFET is immersed in a buffer solution, the element channel current varies with the concentration of hydrogen ions (H^+^), which are generated by the ion layer voltage across the substrate and oxide surface. Therefore, the ISFET is commonly used for H^+^ detection. ISFET development and applications are very diverse (e.g., pH sensors [5], DNA detection [6], glucose monitoring [7], etc.). Figure 1 shows the structure of ISFET [8]. In 1983, J. Van Der Spiegel et al. proposed an extended-gate field-effect transistor (EGFET) [9]. EGFETs are derived from ISFETs. In an ISFET, the metal gate of the MOSFET is replaced with an ion-sensing film, an electrolyte solution, and a reference electrode. By contrast, the EGFET retains the complete MOSFET. The gate of the MOSFET is extended to connect to the metal-sensing film electrolyte solution and the reference electrode. The ISFET has several disadvantages. It can be affected by environmental factors, such as light and temperature, and the device is easily contaminated by electrolytes [10]. The EGFET extends the MOSFET structure by separating the induction region from the MOSFET gate. The sensing area of the MOSFET is connected to the gate through a wire. The EGFET has several characteristics: it is not affected by light or temperature, it is easy to pack, it has a disposable sensor head, and its sensor film is easy to replace. Figure 2 shows the basic cross-section of an EGFET. In previous studies, many researchers have attempted to improve the performance of ISFET devices and compensate for non-ideal effects. Wrege’s research team developed the ISFET chip [11] that was manufactured using a standard 180 nm complementary metal-oxide semiconductor (CMOS) technology. By using the dry test, which is not disturbed by chemical contributions, charges can also be captured in the passivation layer. Manaresi’s research team proposed an 8 × 8 mm chip using dipolycrystalline silicon trimetal 0.35 μm CMOS technology [12]. Epoxy resins are used for bonding line protection. The capillary is connected to the microchamber lateral wall and sealed with insulating glue, allowing the possibility of studying cell dynamics in response to chemicals and cell–cell interactions in real time. Despite this, it does not render the current mode a prevailing solution since the best designs are application-specific, as is usually the case with chemical sensing. ISFETs are also used at the front end of various chemical sensing applications, such as real-time diagnostics and DNA detection [13]. Xu’s research team proposed a CMOS–ISFET-based biomolecular sensing system for DNA detection, which enhanced the charge signal by biasing the ISFET close to the threshold, reducing the sensing area and minimizing the double-layer capacitance of the sensing electrodes [6]. To create low-cost, 3D-printed, highly reproducible, and selective wearable sensors, Kim’s research team investigated the application of 3D-printed conductive carbon-nanofiber–silver-nanowire ink [14]. The inks were integrated into ISFETs for the detection of various electrolytes, such as NH^4+^, K^+^, and Ca^2+^. Massey’s research team investigated organic field-effect transistor (OFET)-based biosensors [15]. The OFET sensors were used to measure saliva, and they were integrated with soft microfluidic channels for fast and reliable quantification of cortisol. Moreover, this process has the advantages of low temperature and low cost.

Ruthenium dioxide (RuO_2_) is a metal oxide with high electrical conductivity, stability, and capacitance that can lead to more charge accumulation in the RuO_2_ sensing window [16,17]. This material is used as a supercapacitor due to its excellent stability in capacitance characteristics [18,19,20]. RuO_2_ is used as a passivation layer for the sensing window on the top metal. In recent years, there have been many studies on the application of pH sensors in ISFETs [11,21,22,23]. However, the combination of enzymes, analytes, and catalysts with microfluidics is rarely discussed. LA is the essential metabolite in the anaerobic metabolism of the human body [24,25]. When there is not enough aerobic breathing energy in the tissue, anaerobic metabolism leads to an elevated lactate concentration [26]. The detection of LA is important for medicine [27], clinical diagnosis [28], athlete care [29], and food analysis [30]. L-lactate dehydrogenase (LDH) and L-lactate oxidase (LOD) can be divided into two enzymes in an enzyme lactate sensor. When LOD is used in the LA sensor, it is easy to cause unstable detection, and it is difficult to miniaturize due to the high oxidation potential and large fluctuation in the oxygen concentration in the solution. LDH exists in blood cells and heart muscle in the body and has high catalytic activity in the auxiliary enzyme nicotinamide adenine dinucleotide (NAD^+^) on LA transformation. The LDH response of the LA biosensor is shown as follows [25]:

(1) $$\mathrm{Lactate}\;+{\;\mathrm{NAD}}^{+}\to \mathrm{Pyruvate}\;+\;\mathrm{NADH}\;+{\;H}^{+}$$

(2) $$\mathrm{NADH}\to {\mathrm{NAD}}^{+}+{\;H}^{+}+2e^{-}$$

In this study, the 0.18 μm CMOS technology provided by Taiwan Semiconductor Manufacturing Company (TSMC) was used to design the MOSFET device and the EGFET sensing window on a chip. The on-chip sensing window (OCSW) was designed with six-layer metals to prevent metal layer collapse. The sensing window size was 931.36 μm × 820.25 μm. First, the RuO_2_ thin film was deposited on the sensing window as passivation layers using the R. F. sputtering system. Then, the lactase was fixed on the RuO_2_ passivation layer, and drops of glutaraldehyde and APTES were added for the immobilization of enzymes to help with functionalization.

In recent years, microfluidic systems have been widely integrated with sensors to enhance analytical performance, real-time detection, and fast response rates [31]. Microfluidic systems have been investigated and applied in many assay areas due to their minimal sample and reagent requirements, high resolution, high sensitivity, low cost, and short analysis time [32]. Thus, in this study, the microfluidic system combined with the EGFET LA sensor was analyzed for dynamic measurement.

## 2. Materials

### 2.1. Materials

The RuO_2_ target (99.95% purity) was purchased from Ultimate Materials Technology Co., Ltd. (Hsinchu City, Taiwan), and the polyethylene terephthalate (PET) substrate was purchased from Zencatec Corporation (Taoyuan City, Taiwan). The LA solution, β-nicotinamide adenine dinucleotide hydrate (NAD^+^), and LDH were from rabbit muscle bought from J. T. Baker Corp. (St. Louis, MO, USA). The γ-aminopropyl triethoxysilane (γ-APTES) and the glutaraldehyde were purchased from Sigma-Aldrich Corp. (St. Louis, MO, USA). The silver paste was purchased from Advanced Electronic Material Inc. (Taipei City, Taiwan), and the epoxy resin was purchased from Sil-More Industrial, Ltd. (Taipei City, Taiwan). The phosphate-buffered saline (PBS, pH 7.0), which was used to mix LA solutions at different concentrations, was purchased from AppliChem GmbH Crop (Darmstadt, Germany), and the deionized water (D. I.) was used for the preparation of aqueous solutions and cleaning of substrates (resistivity = 18.4 MΩ cm^−1^).

### 2.2. The OCSW Integrates the Design of EGFET

**Figure 3:**
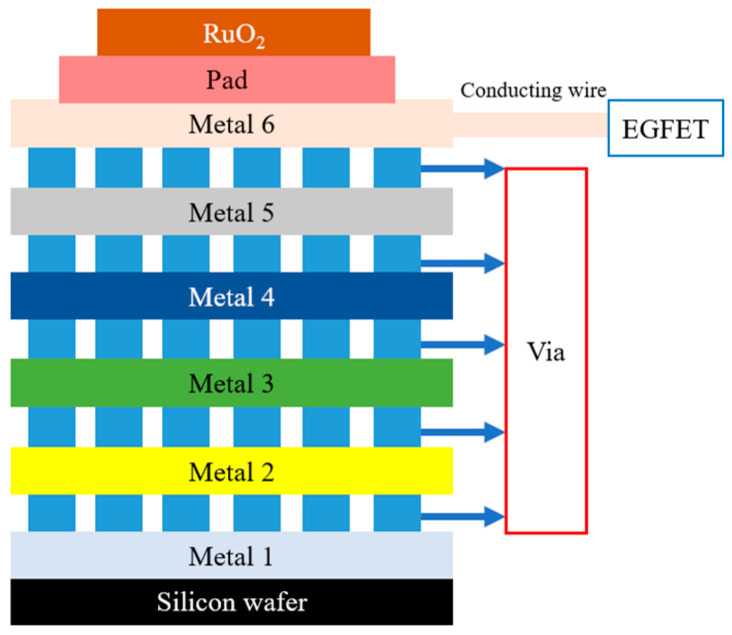
The architecture diagram of OCSW and MOSFET device.

The EGFET consisted of a MOSFET device and an OCSW. The drain source current (I_DS_) of the EGFET followed the basic principle of the MOSFET, and the Gouy–Chapman–Stern model was used in this study [13]. As discussed in the Introduction section, the EGFET is evolved from the ISFET. The EGFET has several characteristics, such as not being affected by light and temperature, being easy to pack, having a disposable sensor head, and having an easy-to-replace sensor film. Thus, in this study, the EGFET was used to analyze the LA sensor. The OCSW was connected to the MOSFET. The architecture diagram of the OCSW and MOSFET device is shown in Figure 3. The sensing window was connected with six layers of metals, and the material of the metals in the TSMC 0.18 μm process was aluminum. The pad was used to bring the top metal into contact with the outside. In this way, the RuO_2_ thin film could be sputtered on the pad. When the RuO_2_ reacted with LA, the response signal was transmitted from metal 6 through the hole conduction to the gate of the MOSFET.

### 2.3. Fabrication of the LA Sensing Film

The LA-sensing film was fabricated on the basis of Chou et al.’s study [33]. First, we used UV-curable glues to seal the outside part of the OCSW, and only the sensing window was spattered with RuO_2_. The RuO_2_ film was deposited on the OCSW using an R.F. sputtering system. Lactase was then titrated onto the sensing window using a micropipette (SPACF0527-01EA, DLAB, City of Industry, CA, USA) under a microscope. Functionalization was facilitated using a glutaraldehyde-to-lactase crosslinking reaction, and APTES was used to immobilize enzymes to help with functionalization [33]. Finally, the chip was left at room temperature for 24 h. After preparation, the film was stored in a 4 °C refrigerator to avoid contamination of the enzyme. Measurements were carried out in two ways: static measurement and dynamic measurement through a microfluidic system. During static measurement, 0.5 μL of LA solution was dropped onto the OCSW. Then, the silver probe was plugged into the LA solution during the measurement. Since the silver probe is lightweight, it can be plugged into the LA solution without losing the surface tension of the LA solution. In the dynamic measurement, a microtube was connected to the OCSW using UV-curable glue packaging to avoid impurities and to prevent other aqueous solutions from damaging the sensing window. 

In the potentiometric biosensor, the response voltage was observed by measuring the variation in hydrogen ion concentration of different LA solutions. The response voltage was determined by the LA concentration, and the sensing regime of the RuO_x_ film of the acquired redox reaction is shown in Formula (3) [34].

(3) $$\mathrm{Ru}{\left(\mathrm{OH}\right)}_{3}↔\;\mathrm{Ru}{\left(\mathrm{OH}\right)}_{2}O^{-}+{\;H}^{+}$$

The potentials of the electrodes were given by a modified version of the Nernst equation, as follows (4) [34,35,36]:

(4) $${E=E}^{0}-\frac{\mathrm{RT}}{F}\mathrm{In}\frac{{\mathrm{Ru}(\mathrm{OH})}_{3}}{{[\mathrm{RuO}}_{2}{][H}^{+}]}{=(E}^{0}-\frac{\mathrm{RT}}{F}\mathrm{In}\frac{{\mathrm{Ru}(\mathrm{OH})}_{3}}{{[\mathrm{RuO}}_{2}]})-\frac{\mathrm{RT}}{F}{\mathrm{In}\;[H}^{+}]$$

where:

E^0^ is the reference electrode potential;
R is the universal gas constant, equal to 8.31 J/(Kmol);
T is the absolute temperature;
F is the Faradays constant, equal to 96485.33 C/mol;
[Ru (OH)_3_] is the activity of RuIII at absolute temperature;
[RuO_2_] is the activity of RuIV at absolute temperature;
H^+^ represents the activity at absolute temperature

Lactase breaks down H^+^ and produces electrons when the LA concentration changes. Therefore, the surface potential of RuO_2_ varies with the concentration of LA. In this study, by observing the changes of I_DS_ of EGFET, the effect of the LA concentration on V_Chem_ could be known.

### 2.4. Sensitivity Measurement of LA Sensing Window

**Figure 4:**
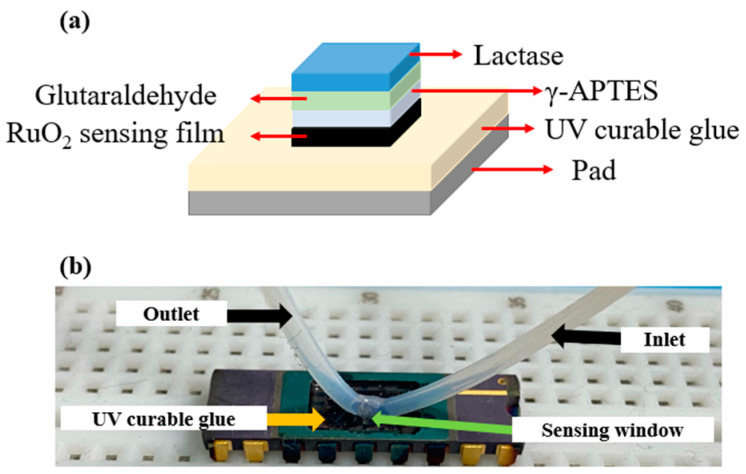
(**a**) The dynamic measurement architecture diagram of EGFET integrated into the OCSW; (**b**) a photo of the dynamic measurement.

**Figure 5:**
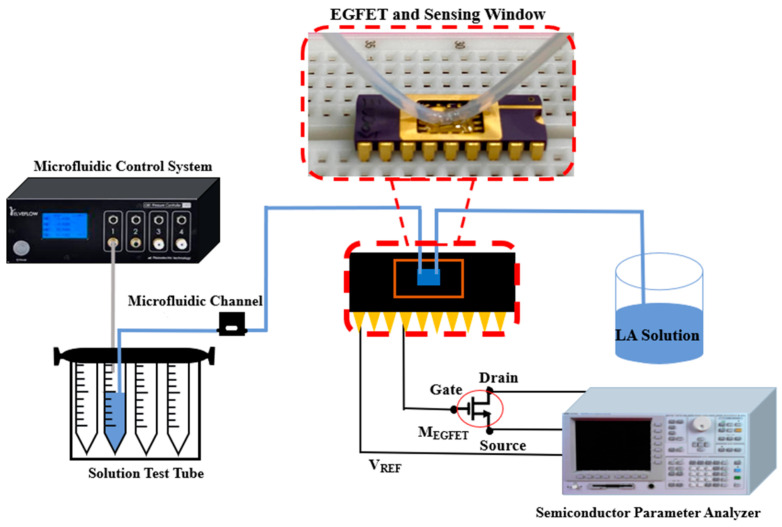
The microfluidic system combined with EGFET measurement.

To test the current–voltage (I–V) characteristics of the LA EGFET, a semiconductor parameter analyzer (SPA, 4156C, Agilent, Santa Clara, CA, USA) was applied. During the measurement, we analyzed the potential difference between the Ag/AgCl reference electrode (RE) and the silver RE according to Chou’s previous work [37]. In this research, silver was used instead of the conventional Ag/AgCl RE for miniaturized measurements. The silver probe was used as a miniature RE, and a silver paste was applied around the silver probe for easy sensing. The silver probe was then plugged into the solution to be measured and finally measured. Figure 4a shows the dynamic measurement architecture diagram of EGFET with the OCSW. Figure 4b shows the photo of the dynamic measurement. To analyze the dynamic measurement, a microtube was connected to the OCSW using UV-curable glue packaging, which prevented contamination of the OCSW by aqueous solutions and damage to lead frames. After that, the source and drain of the MOSFET were connected to the SPA. The silver probe was connected to the SPA as RE and plugged into the LA solution. By measuring the I–V curve, the changes in the I_DS_–V_REF_ curve and I_DS_–V_DS_ curve of the LA EGFET sensor were observed. First, the microfluidic system (OB1 MK3+, Elvesys Microfluidic Innovation Center, Paris, France) was connected to the analyte solution (LA), and the flow rate was adjusted by the flow controller. Then, the liquid was sent into the equipment, and one end of the solution tube was connected to the input of the microfluidic channel. The gate, source, and drain of the EGFET were designed to be connected to an SPA. The complete microfluidic system is shown in Figure 5.

## 3. Results and Discussion

### 3.1. Sensing Analysis of OCSW

**Figure 6:**
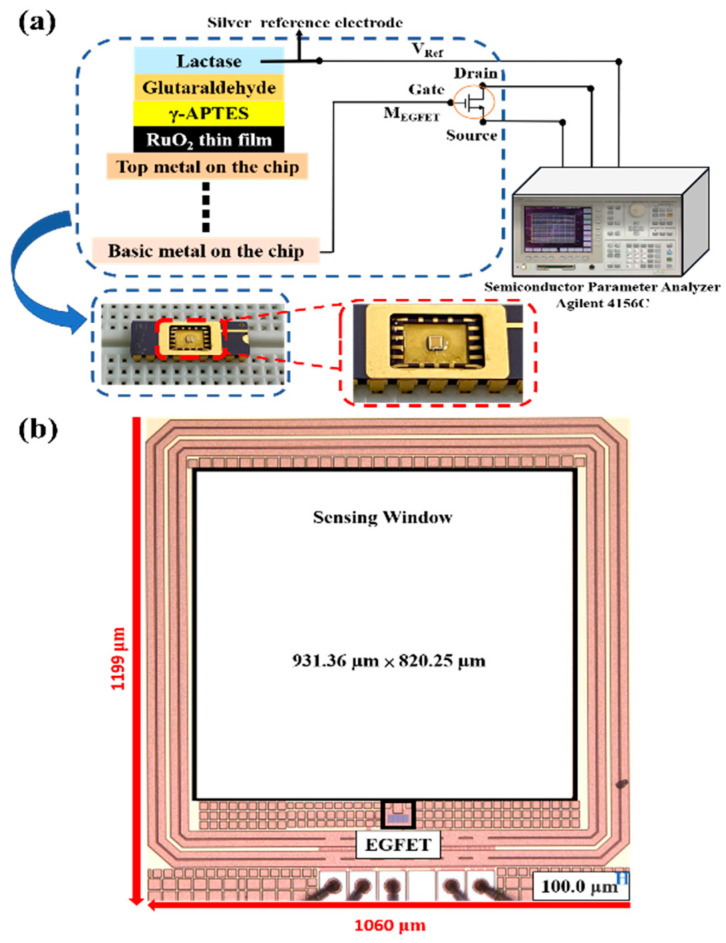
(**a**) Static measurement setup of EGFET with OCSW; (**b**) die photo of EGFET with OCSW.

**Figure 7:**
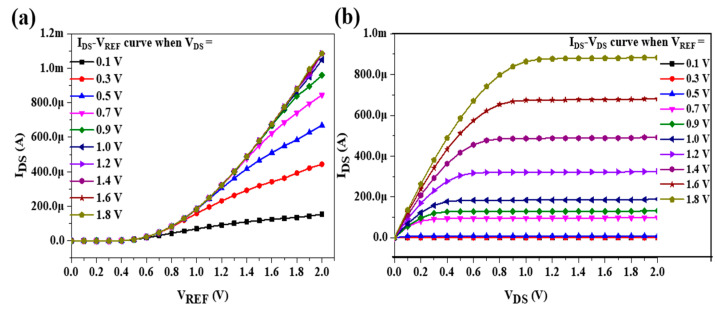
Transmission characteristics of EGFET at different stationary voltages. (**a**) I_DS_–V_REF_ transfer curve; (**b**) I_DS_–V_DS_ transfer curve.

**Figure 8:**
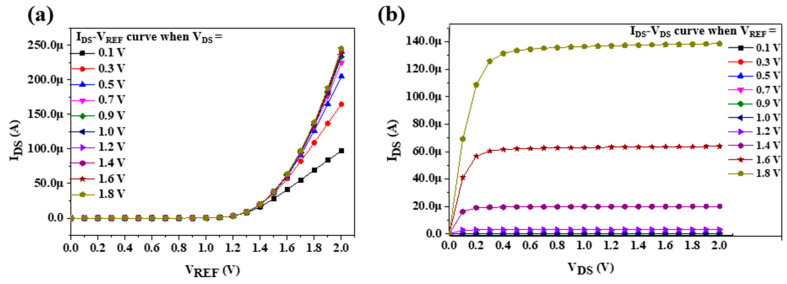
Transmission characteristics of commercial EGFET at different stationary voltages. (**a**) I_DS_-V_REF_ transfer curve; (**b**) I_DS_–V_DS_ transfer curve.

In this study, an EGFET integrated into a sensing window was realized using TSMC 0.18 μm process technology. The chip size was 1199 μm × 1060 μm, and the supply voltage was 1.8 V. Figure 6a shows the static measurement architecture diagram of the EGFET integrated into the OCSW. Figure 6b shows the die photo of the EGFET with the OCSW. The measurement setup for the LA detection is shown in Figure 6a. The SPA was used to observe the transmission characteristics of the EGFET. To analyze the sensing characteristics of the EGFET at different voltages, different values of V_DS_ (0.1 V, 0.3 V, 0.5 V, 0.7 V, 0.9 V, 1.0 V, 1.2 V, 1.4 V, 1.6 V, and 1.8 V) were applied during measurement. V_REF_ was also scanned from 0 to 1.8 V. To verify the function of the self-designed MOSFET, the characteristic curves of commercial MOSFET (CD4007UB) and self-designed MOSFET were measured and compared. The characteristic curves of the I_DS_–V_REF_ with different values of V_DS_ are shown in Figure 7a. The V_DS_ scan range was from 0 to 1.8 V to observe the different saturations of I_DS_ at different values of V_REF_. The transmission curves of the I_DS_–V_DS_ with different values of V_REF_ are shown in Figure 7b. To increase the service life of the components, the saturation current was set below 200 μA because excessive current affects the service life of the equipment [38]. The characteristics of the I_DS_–V_DS_ measured by commercial MOSFET were shown in Figure 8a and the transmission curves of the I_DS_–V_DS_ are shown in Figure 8b. The results show that the performance of the commercial MOSFET was obviously worse than that of the self-designed MOSFET. As shown in Figure 7b, a large I_DS_ was achieved at V_REF_ = 1.6 V when the self-designed MOSFET was applied.

### 3.2. Sensitivity and Linearity of LA Biosensor

**Figure 9:**
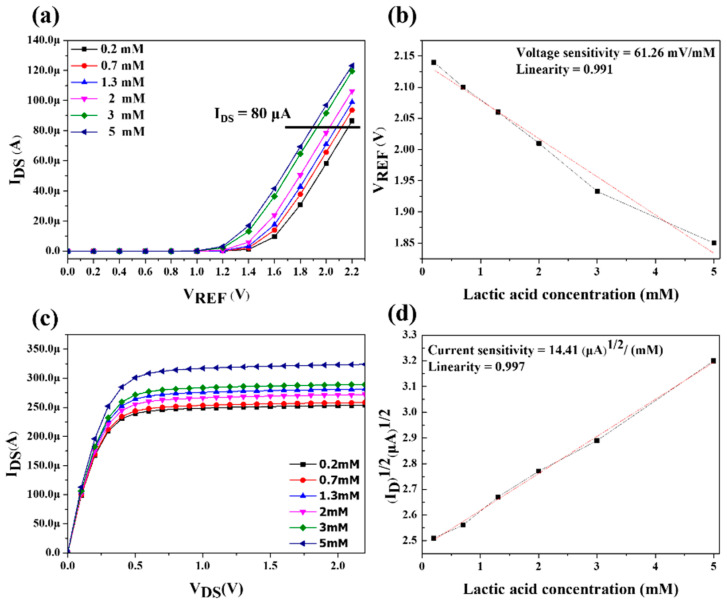
The sensing characteristics of EGFET for LA detection under the static measurement. (**a**) I_DS_–V_REF_ conversion curve, (**b**) voltage sensitivity when I_DS_ = 80 μA, (**c**) I_DS_–V_DS_ transfer curve, and (**d**) current sensitivity.

**Figure 10:**
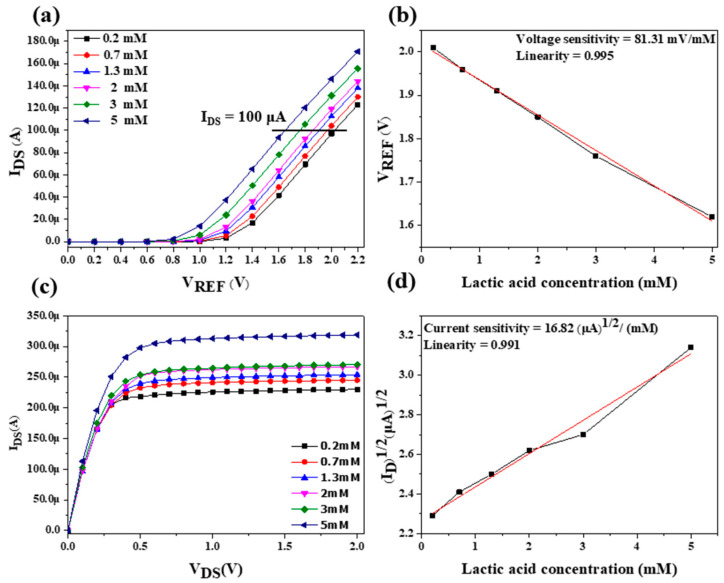
The sensing characteristics of EGFET for LA detection under the dynamic microfluidic measurement. (**a**) I_DS_–V_REF_ conversion curve, (**b**) voltage sensitivity I_DS_ = 100 μA, (**c**) I_DS_–V_DS_ transfer curve, and (**d**) current sensitivity.

The sensitivity characteristics of the LA biosensor were measured at different lactate concentrations. Figure 9a shows the I_DS_–V_REF_ conversion curve by setting I_DS_ = 80 μA, and it can be observed that different concentrations of LA corresponded to different values of V_REF_. Figure 9b shows that the EGFET with a sensing window on the chip for LA detection had a voltage sensitivity of 61.62 mV/mM and linearity of 0.991. Figure 9c shows the I_DS_–V_DS_ transfer curve. The I_DS_ decreased with the decrease in hydrogen ion concentration when the current was saturated. To accurately compute the current sensitivity, V_DS_ = 1 V was chosen. The square root of I_DS_ was described as a function of the LA concentration. As shown in Figure 9d, the current sensitivity was 14.41 (μA)^1/2^/mM, and the linearity was 0.997. The dynamic measurement was performed by immersing the biosensor in different lactate concentrations. The EGFET biosensor with the OCSW based on a microfluidic system was measured by SPA. The I_DS_–V_DS_ transfer curve and I_DS_–V_REF_ conversion curve of the sensing window on the chip were measured at a flow rate of 30 μL/min. Figure 10a shows the I_DS_–V_REF_ conversion curve. By setting I_DS_ = 100 μA, it can be observed that different concentrations of LA correspond to different values of V_REF_. Figure 10b shows the EGFET biosensor under dynamic measurement, and it had a voltage sensitivity of 81.31 mV/mM and a linearity of 0.995. Figure 10c shows the I_DS_–V_DS_ transfer curve. The saturation current decreased as the hydrogen ion concentration decreased. To accurately compute the current sensitivity, V_DS_ = 1 V was chosen, and the square root of I_DS_ was described as a function of the LA concentration, as shown in Figure 10d. The current sensitivity was 16.82 (μA)^1/2^ /mM with a linearity of 0.991. We measured the sensing characteristics of the EGFET for LA detection at different flow rates. The flow rates of 10 μL/min, 20 μL/min, 30 μL/min, 40 μL/min, and 50 μL/min were tested. As shown in Table 1, the biosensor had the best sensing characteristics at a flow rate of 30 μL/min with a voltage sensitivity of 81.31 mV/mM and a linearity of 0.995. Since the diffusion resistance was decreased under dynamic conditions, the sensitivity decreased with the increase in flow rate during the microfluidic measurement experiments [39]. Under the dynamic measurement, the average sensitivity and linearity increased from 61.26 mV/mM and 0.991 to 81.31 mV/mM and 0.995 compared with the static measurement. Therefore, it was demonstrated that the LA sensor has stable sensing characteristics at a flow rate of 30 μL/min. According to previous studies, the thickness of the boundary layer decreases as the flow rate increases and the diffusion of the boundary layer continues to increase [40]. Therefore, the number of enzyme molecules increases, and the diffusion time decreases. When the flow rate was higher than 30 μL/min, the enzyme molecules on the sensing window were disrupted, and the H^+^ ions adsorbed on the surface of the sensing membrane were easily dispersed. Thus, it resulted in a decrease in the sensing characteristics.

### 3.3. Reproducibility

**Figure 11:**
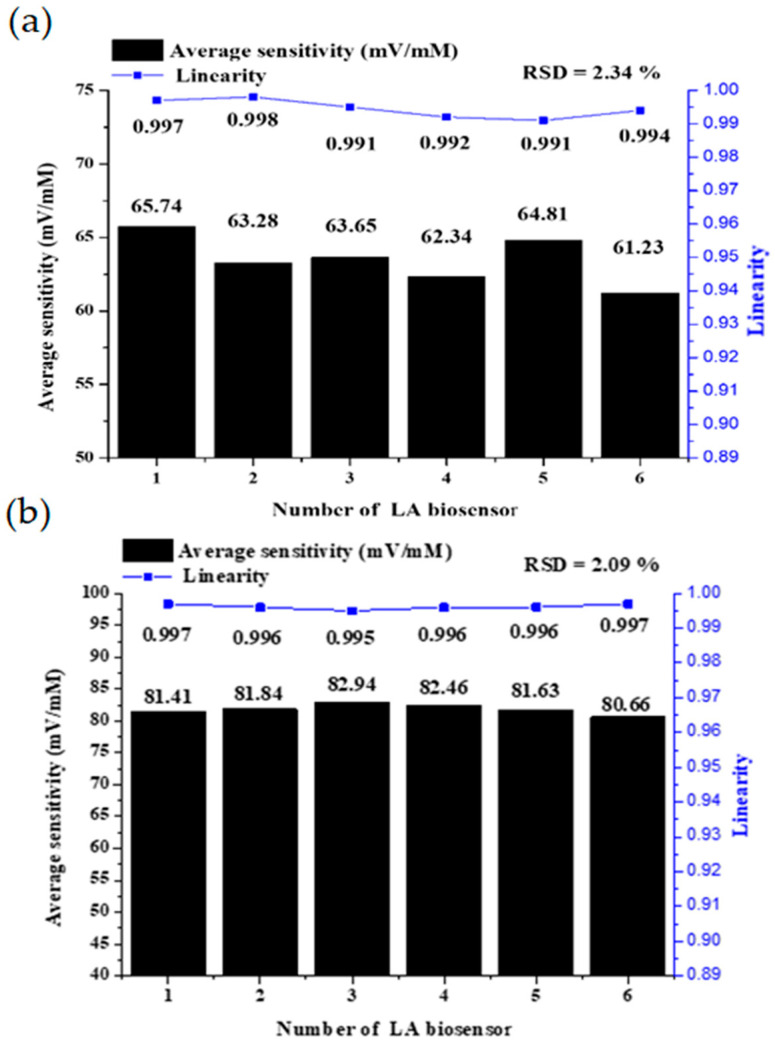
The reproducibility of RuO_2_ LA biosensors. (**a**) Static measurement; (**b**) dynamic microfluidic measurement.

The reproducibility of the RuO_2_ LA biosensor was also measured in this study. Reproducibility is the ability of a biosensor to reconfigure to produce the same response experiments. Reproducibility is characterized by the accuracy and precision of sensors and electronics in biosensors [41]. A biosensor is considered precise when it gives similar results each time the sample is measured, and it is considered accurate when it provides a value close to the average over multiple measurements of the sample. The reproducibility of static and dynamic measurements of the RuO_2_ LA biosensor is shown in Figure 11. The six biosensors were prepared simultaneously using the same method; the average sensitivity and the linearity were measured, and the above steps were repeated 10 times. The relative standard deviation (RSD) of the RuO_2_ LA biosensor was 2.34% with static measurement and 2.09% with dynamic microfluidic measurement. The calculation method was the ratio of standard deviation to the arithmetic mean, where S is the standard deviation of average sensitivity, X is the mean value of average sensitivity, and the RSD of the average sensitivities is determined. We used six values of average sensitivity to find the mean and standard deviation; the formula is as follows [42]:

(5) $$\mathrm{RSD}=\frac{S}{X}\times \;100\%$$

Table 2 shows the comparisons of static and dynamic measurements. The static and dynamic measurements had good stability. For static measurement, 50 mL of LA solution was required; however, for dynamic measurement, 10 mL of LA solution was required, significantly reducing the amount of solution used. In terms of preparation, dynamic measurement requires special packaging and a microfluidic combination of the chip. Thus, the cost of the dynamic measurement system is higher than that of the static measurement. Overall, dynamic measurement has better sensitivity, linearity, and RSD compared with static measurement.

### 3.4. Sensing Characteristics Analysis of the Standard Sensor

**Figure 12:**
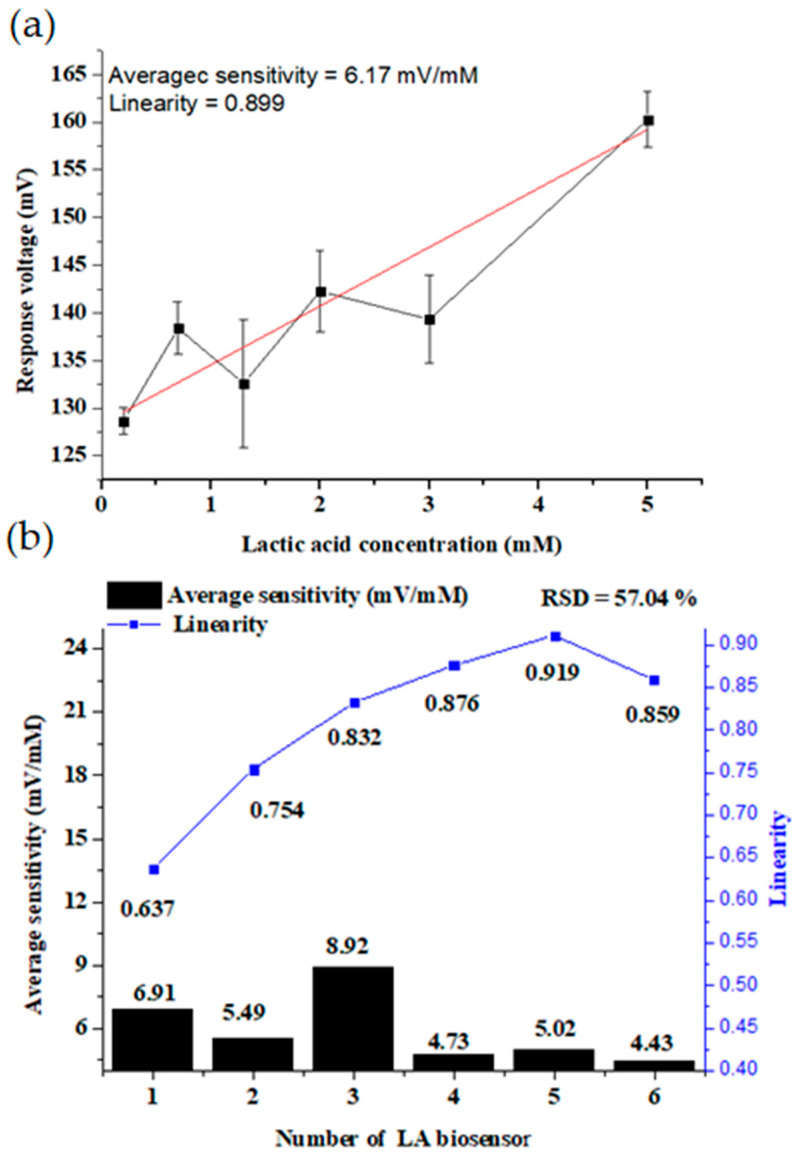
The (**a**) average sensitivity and linearity; (**b**) reproducibility of the standard sensor.

To verify the authenticity of the measurement results with and without the lactate/RuO_2_ architecture, we used sensors without RuO_2_ film and lactate modification as standard sensors and measured their average sensitivity, linearity, and repeatability. To analyze our sensor without sensing film and lactase, we followed the approach proposed in [43], in which we used the sensor without RuO_2_ film and lactase modification as the standard sensor. This standard sensor was immersed in different concentrations of lactic acid solution. Then, the sensing characteristics were obtained, as shown in Figure 12. The average sensitivity and linearity were 6.17 mV/mM and 0.899, respectively; the RSD was 57.04%, and the results were poor. Lactase, through the hydrogen ions, accumulates on the surface of the sensing film [43]. Different LA concentrations produce different response voltages. Three kinds of adsorbed states, O^−^, OH^2+^, and OH, were generated on the sensing film when it was immersed in the solution. The RuO_2_ lactate biosensor detected the H^+^ ion concentrations to obtain the response voltage. Therefore, the enzymatic LA sensors cannot respond without RuO_2_, lactase, or LA solution for measurement.

Table 3 shows the comparisons of the characteristics of diverse LA biosensors. Madden et al. showed slower electron transfer behavior due to chitosan and lactate oxidase modification, which could be attributed to the nonconductive chitosan covering an area of the Pt-LSG electrode [44]. Thus, it slowed down the diffusion of redox substances to the electrode. It can be used to measure saliva or dilute serum analysis for low-cost diagnostic potential. Chou et al. investigated the remote monitoring of glucose and lactate. To ensure the stability of the LA biosensor, graphene oxide (GO) was used to increase the surface area and improve biocompatibility and magnetic beads (MB_s_) to enhance biocompatibility and physicochemical stability. The average sensitivity was 45.4 mV/mM, the response time was 21 s, and the drift rate was 12.1 mV/h [45]. Chou et al. studied IGZO LA biosensors and improved the average sensitivity through GO and MBs [46]. First, the sensing characteristics of different proportions of modifiers added to the LA biosensor were analyzed. Then, the best ratio was combined with a microfluidic system to analyze the sensing changes of the LA biosensor at different flow rates. When the flow rate was 25 μL/min, the average sensitivity and linearity were 70.37 mV/mM and 0.967, respectively. Diallo et al. used a pH-based ElecFET biosensor and bovine serum albumin (BSA) in glutaraldehyde crosslinking to study and compare the ElecFET responses obtained with and without hydrogen peroxide [47]. H_2_O_2_ is an enzyme used for the detection of H_2_ and O_2_ biomolecules. The detection is achieved by functionalizing the surface with particular enzymes (LOX). Compared with previous studies, our proposed biosensor had better sensitivity and linearity. An EGFET with an OCSW has the advantages of miniaturized size, low cost, and good sensing characteristics. A key advantage of using EGFET biosensors is the direct transfer of physical properties to detect the analyte. This significantly reduces the manufacturing complexity and expense. In addition, the use of electrolytes provides large capacitance to enable low power operation, printability, and flexibility. Aqueous electrolytes in the liquid state allow the receptor to be functionalized in a dense layer directly on the gate or channel of the transistor to obtain higher sensitivities. Aqueous electrolytes provide low electrical conductivity, increase ionic conductivity, and increase biocompatibility [48]. Avci’s team developed the EGFET pH micro-sensor for fast pH detection; after measuring pH 5 to pH 12, the response was accurate. The addition of polypyrrole enhances EGFET pH biocompatible, allows high conductivity, and provides easy polymerization capabilities to potential sensors. EGFET microsensors can be used for pH measurement in bioanalytical applications with advantages such as miniaturization, easy integration into different microsystems, stability during environmental changes, high conductivity, and sensitivity [49]. There are great prospects for the future development of the EGFET. To solve the packaging problem, UV-curable glues can be used to isolate moisture and chemical contamination [50]. In this study, it was learned that the OCSW is easy to clean and can be combined with the microfluidic system for measurement. In the future, it can be applied to wearable devices, which can detect the physical condition of the body in tiny amounts.

## 4. Conclusions

In this study, an EGFET lactic acid biosensor was fabricated by sputtering a lactase /RuO_2_ sensing film onto an OCSW. In the static measurement, the biosensor had an average sensitivity of 61.26 mV/mM and a linearity of 0.991. In the dynamic measurement, the biosensor had an average sensitivity of 81.31 mV/mM and a linearity of 0.995. The biosensor had better sensing characteristics due to the promotion of the catalytic reaction and the reduction in diffusion resistance. The best sensitivity and linearity were 81.31 and 0.995 when the flow rate was 30 μL/min. In addition to the characteristics of the EGFET, the OCSW measurement increased the ionic conductivity and increased biocompatibility, with good sensing characteristics. Therefore, it is suitable for the detection of LA. The reproducibility was measured to prove the precision and accuracy of the device, and the RSD was 2.34%. Moreover, this sensor has the advantages of miniaturized size and low cost. Thus, the proposed biosensor can be integrated with a wearable device for LA detection in the human body. In the future, serum can be used as the test sample. In addition, the sensing film can also be modified by GO or nanoparticles to improve the sensing ability of LA biosensors.

## Figures and Tables

**Table 1:** The response voltages under different flow rates.

Flow Rate (μL/min)	Voltage Sensitivity (mV/mM)	Linearity
0	61.26	0.991
10	62.32	0.993
20	72.84	0.992
30	81.31	0.995
40	67.43	0.985
50	52.29	0.978

**Table 2:** Comparisons of static and dynamic measurements.

Measurements	Sensitivity (mV/mM)	Linearity	RSD%	Cost	Solution Amounts
Static	61.26	0.991	2.34	lower	more
Dynamic	81.31	0.995	2.09	higher	less

**Table 3:** Comparisons of the sensing characteristics for diverse LA biosensors.

Sensing Equipment	Sensing Films	Linear Range (mM)	Sensitivity (mV/mM)	Linearity	RSD%	Reference
EGFET (static)	Lactate/APTES/RuO_2_	0.2–5	61.26	0.991	2.34	In current study
EGFET (dynamic)	Lactate/APTES/RuO_2_	0.2–5	81.31	0.995	N/A	In current study
LSG	LO_x_/Pt/CS	0.2–3	35.8 μA/mM/cm^2^	0.999	2.9	[44] 2022
LT1167	Lactate/MB_s_/ GPTS/GO/NiO	0.2–3	46.70	0.998	N/A	[45] 2018
LT1167	Lactate/MBs/IGZO	0.3–3	70.37	0.967	N/A	[46] 2018
ElecFET	LOD/Si_3_N_4_	1–6	20.00	N/A	N/A	[47] 2013

## Data Availability

Not applicable.

## References

[1] Jing K.H., Arshad M.K.M.d., Huda A.R.N., Ruslinda R., Gopinath S.C.B., Nuzaihan M.N.M., Ayub R.M., Fathil M.F.M., Othman N., Hashim U. Gate Dielectric Scaling in MOSFETs Device. Proceedings of the 2016 AIP Conference.

[2] Liu J., Ohsato H., Wang X., Liao M., Koide Y. (2016). Design and Fabrication of High-Performance Diamond Triple-Gate Field-Effect Transistors. Sci. Rep..

[3] Kahng D. (1976). A Historical Perspective on the Development of MOS Transistors and Related Devices. IEEE Trans. Electron Devices.

[4] Bergveld P. (1970). Development of an Ion-sensitive Solid-State Device for Neurophysiological Measurements. IEEE Trans. Biomed. Eng..

[5] Goda T. (2021). Chemically Induced pH Perturbations for Analyzing Biological Barriers Using Ion-Sensitive Field-Effect Transistors. Sensors.

[6] Xu G., Abbott J., Ham D. (2016). Optimization of CMOS-ISFET-Based Biomolecular Sensing: Analysis and Demonstration in DNA Detection. IEEE Trans. Electron Devices.

[7] Zhao S., Shi C., Hu H., Li Z., Xiao G., Yang Q., Sun P., Cheng L., Niu W., Bi J. (2020). ISFET and Dex-Agnps Based Portable Sensor for Reusable and Real-Time Determinations of Concanavalin A and Glucose on Smartphone. Biosens. Bioelectron..

[8] Chaudhary R., Sharma A., Sinha S., Yadav J., Sharma R., Mukhiya R., Khanna V.K. Fabrication and Characterization of Al Gate N-MOSFET, on-chip Fabricated with Si_3_N_4_ ISFET. Proceedings of the 19th International Symposium on VLSI Design and Test.

[9] Spiegel J.v.d., Lauks I., Chan P., Babic D. (1983). The Extended Gate Chemically Sensitive Field Effect Transistor as Multi-Species Microprobe. Sens. Actuators.

[10] Chou J.C., Chen C.W. (2009). Fabrication and Application of Ruthenium-Doped Titanium Dioxide Films as Electrode Material for Ion-Sensitive Extended-Gate FETs. IEEE Sens. J..

[11] Wrege R., Peter C., Wesling B.N., Rambo C.R., Schneider M.C., Galup-Montoro C. (2021). A CMOS Test Chip with Simple Post-Processing Steps for Dry Characterization of ISFET Arrays. IEEE Sens. J..

[12] Manaresi N., Romani A., Medoro G., Altomare L., Leonardi A., Tartagni M., Guerrieri R. (2003). A CMOS Chip for Individual Cell Manipulation and Detection. IEEE J. Solid-State Circuits.

[13] Miscourides N., Georgiou P. (2019). ISFET Arrays in CMOS: A Head-to-Head Comparison Between Voltage and Current Mode. IEEE Sens. J..

[14] Kim T., Bao C., Hausmann M., Siqueira G., Zimmerman T., Kim W.S. (2019). 3D Printed Disposable Wireless Ion Sensors with Biocompatible Cellulose Composites. Adv. Electron. Mater..

[15] Massey R.S., Prakash R. (2022). A Low-Temperature-Processed, Soft-Fluidic OEGFET Saliva Aptasensor for Cortisol. IEEE J. Flex. Electron..

[16] Tseng S.C., Wu T.Y., Chou J.C., Liao Y.H., Lai C.H., Chen J.S., Huang M.S. (2016). Research of Non-Ideal Effect and Dynamic Measurement of The Flexible-Arrayed Chlorine Ion Sensor. IEEE Sens. J..

[17] Sadig H.R., Li C. (2020). Applying a Novel Polymeric Precursor Derived by Capillary-Gravitational Coating in Fabrication of Nanostructured Tri- Metal Oxide-Based pH Sensing Electrode. IEEE Sens. J..

[18] Zhang Q., Gu D., Li H., Xu Z., Sun H., Li J., Shen L. (2021). Energy Release From RuO_2_//RuO_2_ Supercapacitors Under Dynamic Discharge Conditions. Electrochim. Acta.

[19] Asbani B., Robert K., Roussel P., Brousse T., Lethien C. (2021). Asymmetric Mmicro-Supercapacitors Based on Electrodeposited RuO_2_ and Sputtered VN Films. Energy Storage Mater..

[20] Huang M.J., Chen W.H., Cheng C., Chen S.R., Lin J.Y., Yang C.R. (2021). Integration of RuO_2_ /Conductive Fiber Composites within Carbonized Micro-Electrode Array for Supercapacitors. J. Alloys Compd..

[21] Wang L., Li L., Zhang T., Liu X., Ao J.P. (2018). Enhanced pH Sensitivity of AlGaN/GaN Ion-Sensitive Field Effect Transistor with Al_2_O_3_ Synthesized by Atomic Layer Deposition. Appl. Surf. Sci..

[22] Singh K., Pang S.T., Pan T.M. (2021). Amorphous ZnSn_x_O_y_ Fabricated at Room-Temperature for Flexible pH-EGFET Sensor. IEEE Trans. Electron Devices.

[23] Elyasi A., Fouladian M., Jamasb S. (2018). Counteracting Threshold-Voltage Drift in Ion-Selective Field Effect Transistors (ISFETs) Using Threshold-Setting Ion Implantation. IEEE J. Electron Devices Soc..

[24] Hickey D.P., Reid R.C., Milton R.D., Minteer S.D. (2016). A Self-Powered Amperometric Lactate Biosensor Based on Lactate Oxidase Immobilized in Dimethylferrocene-Modified LPEI. Biosens. Bioelectron..

[25] Rathee K., Dhull V., Dhull R., Singh S. (2016). Biosensors Based on Electrochemical Lactate Detection: A Comprehensive Review. Biochem. Biophys. Rep..

[26] Todd J.J. (2014). Lactate: Valuable for Physical Performance and Maintenance of Brain Function During Exercise. Biosci. Horiz. Int. J. Stud. Res..

[27] Chiu T.K., Lei K.F., Hsieh C.H., Hsiao H.B., Wang H.M., Wu M.H. (2015). Development of a Microfluidic-Based Optical Sensing Device for Label-Free Detection of Circulating Tumor Cells (CTCs) Through Their Lactic Acid Metabolism. Sensors.

[28] Tsai Y.C., Chen S.Y., Liaw H.W. (2007). Immobilization of Lactate Dehydrogenase Within Multiwalled Carbon Nanotube-Chitosan Nanocomposite for Application to Lactate Biosensors. Sens. Actuators B Chem..

[29] Parra A., Casero E., Vázquez L., Pariente F., Lorenzo E. (2006). Design and Characterization of a Lactate Biosensor Based on Immobilized Lactate Oxidase onto Gold Surfaces. Anal. Chim. Acta.

[30] Mazzei F., Azzoni A., Cavalieri B., Botre F., Botre C. (1996). A Multi-Enzyme Bioelectrode for The Rapid Determination of Total Lactate Concentration in Tomatoes, Tomato Juice and Tomato Paste. Food Chem..

[31] Salim A., Lim S. (2018). Review of Recent Metamaterial Microfluidic Sensors. Sensors.

[32] Booth J.C., Orloff N.D., Mateu J., Janezic M., Rinehart M., Beall J.A. (2010). Quantitative Permittivity Measurements of Nanoliter Liquid Volumes in Microfluidic Channels to 40 GHz. IEEE Trans. Instrum. Meas..

[33] Nien Y.H., Su Z.Y., Ho C.S., Chou J.C., Lai C.H., Kuo P.Y., Kang Z.X., Dong Z.X., Lai T.Y., Wnag C.H. (2020). The Analysis of Potentiometric Flexible Arrayed Urea Biosensor Modified by Graphene Oxide and γ-Fe_2_O_3_ Nanoparticles. IEEE Trans. Electron Devices.

[34] Singh K., Lou B.S., Her J.L., Pang S.T., Pan T.M. (2019). Super Nernstian pH Response and Enzyme-Free Detection of Glucose Using Sol-Gel Derived RuO_x_ on PET Flexible-Based Extended-Gate Field-Effect Transistor. Sens. Actuators B Chem..

[35] Lonsdale W., Shylendra S.P., Brouwer S., Wajrak M., Alameh K. (2018). Application of Ruthenium Oxide pH Sensitive Electrode to Samples with High Redox Interference. Sens. Actuators B Chem..

[36] Kurzweil P. (2009). Precious Metal Oxides for Electrochemical Energy Converters: Pseudocapacitance and pH Dependence of Redox Processes. J. Power Source.

[37] Chou J.C., Tsai Y.H., Chen C.C. (2008). Development of a Disposable All-Solid-State Ascorbic Acid Biosensor and Miniaturized Reference Electrode Fabricated on Single Substrate. IEEE Sens. J..

[38] Kuo P.Y., Chen Y.Y. (2021). A Novel Low Unity-Gain Frequency and Llow Power Consumption Instrumentation Amplifier Design for RuO₂ Uric Acid Biosensor Measurement. IEEE Trans. Instrum. Meas..

[39] Chou J.C., Ye G.C., Wu D.G., Chen C.C. (2012). Fabrication of the Array Chlorine Ion Sensor Based on Microfluidic Device Framework. Solid-State Electron..

[40] Samphao A., Butmee P., Jitcharoen J., Svorc L., Raber G., Kalcher K. (2015). Flow-Injection Amperometric Determination of Glucose Using a Biosensor Based on Immobilization of Glucose Oxidase onto Au Seeds Decorated on Core Fe_3_O_4_ Nanoparticles. Talanta.

[41] Morales M.A., Halpern J.M. (2018). Guide to Selecting a Biorecognition Element for Biosensors. Bioconjug. Chem..

[42] Chou J.C., Lin S.H., Lai T.Y., Kuo P.Y., Lai C.H., Nien Y.H., Su T.Y. (2020). A Facile Fabrication of a Potentiometric Arrayed Glucose Biosensor Based on Nafion-GOx/GO/AZO. Sensors.

[43] Chou J.C., Lee K.T., Lai C.H., Kuo P.Y., Nien Y.H., Huang Y.H., Kang Z.X. (2022). Novel Potentiometric Non-Enzymatic Ascorbic Acid Sensor Based on Molybdenum Oxide Film and Copper Nanoparticles. IEEE Sens. J..

[44] Madden J., Vaughan E., Thompson M., Riordan A.O., Galvin P., Lacopino D., Teixeira S.R. (2022). Electrochemical Sensor for Enzymatic Lactate Detection Based on Laser-scribed Graphitic Carbon Modified with Platinum, Chitosan and Lactate Oxidase. Talanta.

[45] Chou J.C., Yan S.J., Liao Y.H., Lai C.H., Wu Y.X., Wu C.Y. (2018). Remote Detection for Glucose and Lactate Based on Flexible Sensor Array. IEEE Sens. J..

[46] Chou J.C., Chen H.Y., Liao Y.H., Lai C.H., Yan S.J., Wu C.Y., Wu Y.X. (2018). Sensing Characteristic of Arrayed Flexible Indium Gallium Zinc Oxide Lactate Biosensor Modified by GO and Magnetic Beads. IEEE Trans. Nanotechnol..

[47] Diallo A.K., Djeghlaf L., Mazenq L., Launay J., Sant W., Temple-Boyer P. (2013). Development of pH-Based ElecFET Biosensors for Lactate Ion Detection. Biosens. Bioelectron..

[48] Wang G.Y., Lian K., Chu T.Y. (2021). Electrolyte-Gated Field Effect Transistors in Biological Sensing: A Survey of Electrolytes. IEEE J. Electron Devices Soc..

[49] Avci I., Oğuz M., Şen M. An Extended Gate Field Effect Transistor (EGFET) pH Microsensor. Proceedings of the 2021 Medical Technologies Congress.

[50] Wägli P., Homsy A., De Rooij N.F. (2011). Norland Optical Adhesive (NOA81) Microchannels with Adjustable Wetting Behavior and High Chemical Resistance Against a Range of Mid-Infrared-Transparent Organic Solvents. Sens. Actuators B Chem..

